# Reputable Manufacturing Pharmacists Do Not Furnish Emmenagogues for Immoral Purposes

**Published:** 1914-01

**Authors:** 


REPUTABLE MANUFACTURING PHARMACISTS DO NOT FURNISH EMMENAGOGUES FOR IMMORAL PURPOSES.—Recently one of the leading manufacturing pharmaceutical houses received a letter upon the letterhead of a retail druggist, but signed by another name followed by the word "druggist." The person signing the letter may have been a clerk or successor of the druggist. The letter was as follows:

"There is practically no sale for your Emmenagogue Improved Pills, as few ladies know anything about them, and we can give no advice, as we know nothing about them ourselves as to dose, etc. Please let us know by return mail and tell us how to use, dose, etc."

Reply was made to the pharmacist whose name was on the letterhead, and was as follows:

"We have our doubts about Mr. ——— being a druggist, for we cannot imagine any druggist not knowing that it is not only immoral, but criminal, to sell an emmenagogue except upon a physician's prescription. We believe that every druggist who sells an emmenagogue direct to the consumer is put upon his notice that it will be used for an immoral and criminal purpose. Emmenagogues on our list are intended exclusively for the prescription trade and we never knowingly sell them for popular use or to be recommended and resold as remedies for female complaints, etc."

---

A not uncommon cause of persistent pain in the knee is bursitis sartorius, semimembranosus beneath the inner hamstring tendon insertions.—*American Journal of Surgery.*

